# Correlation between follicle dimensions recorded by patients at home (SOET) versus ultrasound performed by professional care providers.

**Published:** 2017-09

**Authors:** L Dalewyn, E Deschepper, J Gerris

**Affiliations:** Women’s Clinic, University Hospital Ghent, De Pintelaan 185, 9000 Ghent,Belgium; Biostatistics Unit, Ghent University, De Pintelaan 185, 9000 Ghent, Belgium

**Keywords:** self-operated vaginal sonography, follicular growth monitoring, ART cycles, SOET, patient friendly, telemonitoring

## Abstract

**Introduction:**

Serial measurements of the number of follicles and their growth by ultrasound is a standard way of monitoring fertility treatments using controlled ovarian stimulation. This is stressful for both the patient and the professional. Self-operated endovaginal telemonitoring (SOET) is more patient friendly and less time-consuming.

**Aim of the study:**

The goal of the study is to see if there’s a correlation in the number of follicles and in two- dimensional growth between recordings made using SOET versus measurements performed by a professional sonographer.

**Results:**

Three different ultrasound moments were recorded and compared in a total of 15 women. At time A an ultrasound was performed by the patient at home using SOET at the decision time of triggering. At time B an ultrasound was also recorded by the patient, 24 hours later. At time C an ultrasound was performed by a physician using a high end ultrasound device immediately prior to oocyte retrieval, 12 hours later than time B. The correlation in number and two-dimensional size between the different measurement moments was calculated. There is an excellent correlation in follicle count between time B and C. The difference in mean two-dimensional size between different measurement moments was not statistically significant.

**Conclusion:**

SOET ultrasound correlates well with ultrasound performed by a professional in number of follicles. SOET is a good alternative for monitoring controlled ovarian stimulation in a well-defined population group of normal responders, especially near the end of the ovarian stimulation.

## Introduction

Fertility treatments with ovarian stimulation are traditionally monitored by serial sonographic measurements of follicular growth and by serial measurements of serum hormone values. This is necessary in the follow-up of IVF/ICSI cycles to adjust the dose of stimulation medication and to determine the timing of oocyte retrieval. Especially monitoring follicular number and size is of great importance for timing the moment of triggering and for early detection of OHSS. Because of this, fertility treatments are time-consuming for the patients as well as for the professional care providers. Clinical research is being conducted in order to simplify the monitoring of stimulated cycles and to minimize the burden for the patient.

For example the patient can perform the ultrasound herself, at home, in fact anywhere anytime.

The idea is to learn a patient to make their sonographies themselves, after proper instruction by a professional. The patient uses a portable device with an endovaginal probe, allowing registration of real time images under direct visual inspection of the patient or her partner. When she is satisfied with the images she screens both ovaries (each 30 seconds) and the uterus with endometrial thickness (15 seconds). These images are sent with a cloud-application to the physician who can store and analyse the images. The physician can measure the number of follicles and follicular dimensions and the endometrial thickness. Instructions for the patient including dose adjustment of gonadotropins, timing of HCG triggering and timing of oocyte retrieval are sent back to the patient with the cloud application. (Gerris et al., 2009; 2016)

Previous research by our group showed that home sonography is not inferior to traditional follow-up in terms of numbers of oocytes, numbers of mature metaphase II oocytes, conceptions and ongoing pregnancies (Gerris et al., 2014). This method of monitoring IVF/ICSI cycles has a lot of benefits: less transportation costs, less time consuming, more flexible, ... (Gerris et al., 2009; 2016). However, concerns have to be addressed regarding image quality. This study aimed to compare the quality of SOET images and measurements against the traditional way of monitoring.

Therefore, we studied the correlation between the number of follicles and growth recorded by SOET (self-operated endovaginal telemonitoring) and the measurements performed by a professional sonographer. The study compares the number and size of follicles at the end of ovarian stimulation for IVF/ICSI between observations based on home sonographic recordings by the patient using low-end home sonographic equipment linked to a specific cloud application versus observations using high-end sonographic equipment by the physician performing oocyte retrieval.

## Methods

In this pilot study, a total of fifteen women were included in the study, all women were treated with the home sonographic device (SOET). They all had previously undergone IVF/ICSI stimulation(s) and were regarded as ‘normal’ responders. A standard treatment protocol was used. After suppression using combined oral contraception, patients were stimulated using 0,1mg GnRH agonist (triptorelin acetate; Decapeptyl®, Ferring Pharmaceuticals) per day for 7 days and follitropin alfa (Gonal-F®, Merck Serono) with an initial stimulation dose as a function of serum antimullerian hormone (La Marca et al., 2012). All patients received Pregnyl® 5000 IE subcutaneously as trigger for the final oocyte maturation. Oocyte puncture, embryo culture (up to day 5 blastocysts) and embryo transfer, as well as luteal support and embryo cryopreservation are all carried out according to international guidelines (Magli et al., 2008).

The home sonography is performed in a standardized manner. The patient has to scan both ovaries and the uterus during respectively 30 seconds for each ovary and 15 seconds for the uterus in the following order: right ovary, uterus, left ovary. The patient can practice for an indefinite time frame before making the actual recordings. These images are sent by a cloud application to the physician. The following variables are measured by the physician: the number of follicles, the 2D dimensions and the thickness of the endometrial lining.

Three different ultrasound moments were chosen for recordings and compared in the study. The first measurement is the standard ‘last’ ultrasound 36 hours before the ovarian pick-up, performed at home by the patient with SOET. This is the moment of HCG injection to trigger ovulation (Pregnyl®) (time A). A second, supernumerary, ultrasound, also recorded by the patient at home, was performed 24 hours after the injection with Pregnyl® (Time B). Both images were sent using the cloud application. The last ultrasound was performed by a physician, with a high technological device (Medison SonoAce X8, Samsung, Seoul, Korea), just before ovarian pick-up, 36 hours after the triggering time (Time C). This ultrasound was performed by the physician who performed the ovarian pick-up. The time difference between time B and C is 12 hours.

The physician who performed the ultrasound at time C was blinded and did not know the result of the previous ultrasounds at time A and B. The measurements of the recordings at time B were clustered and analysed by another physician on one single day. This was to minimize the intra- and interobserver variability.

## Results

**Figure 1:**
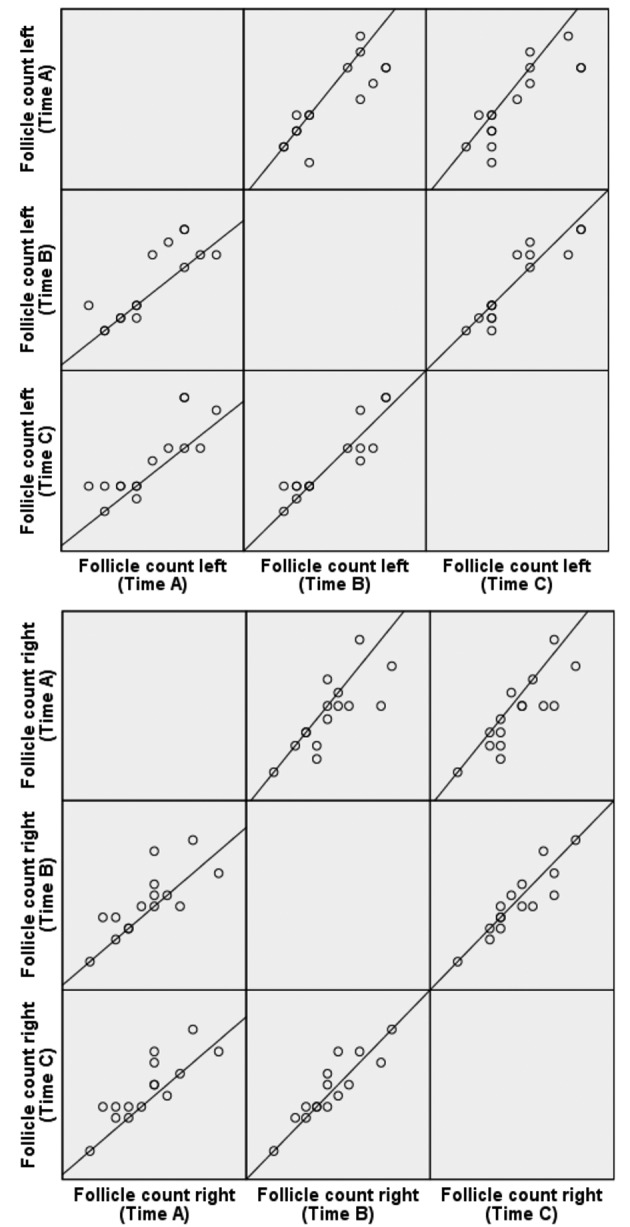
— Scattermatrix showing, respectively for the left and right ovary, the number of follicles on the three measurement moments in relation to each other.

Figure 1 is a graphical representation showing a scattermatrix composed of 6 scatterplots respectively for the left and right ovary. The number of follicles on the 3 measurement times is visually shown in relation to each other. If the number of follicles would be identical per patient per measurement time, all would be in the same line (y=x). The visual similarity is the greatest between time B and C. This seems logical since there is only 12 hours difference in time of recording. The number of follicles measured earlier in time seems to be somewhat lower than the ones measured later in time. In conclusion, the number of follicles correlates well between SOET and conventional 2D ultrasound.

The Intraclass Correlation Coefficient (ICC) is excellent between the different groups in accordance with the classification of Cicchetti and Sparrow (1981): ICC>0,75 “excellent”, ICC between 0,60 and 0,74 “good”, ICC between 0,40 and 0,59 “fair”, ICC < 0,40 “poor”. Nevertheless, this has to be interpreted with caution because the confidence interval is quite large, especially when comparing the measurements with the biggest time interval between the sonographies. There is an excellent correlation between time B and time C (Table I).

**Table I:** Intraclass Correlation Coefficient (ICC) with 95% coincidence interval (CI) between the different measurement times.

	LEFT			RIGHT		
	ICC	LL 95% CI	UL 95% CI	ICC	LL 95% CI	UL 95% CI
3 measurement times	,832	,639	,936	,815	,612	,928
						
Time A vs. time C	,773	,359	,923	,788	,351	,930
Time A vs. time B	,795	,465	,928	,750	,406	,908
Time B vs. time C	,918	,778	,972	,904	,745	,967

The mean follicle count recorded per measurement moment compared between the three measurements, after correcting for side, is not significant different (p=0,051) on the basis of a mixed model analysis with random intercept for patient. Comparing mean follicle count between left and right ovary shows no statistically significant difference, after correction for measurement moment.

**Figure 2:**
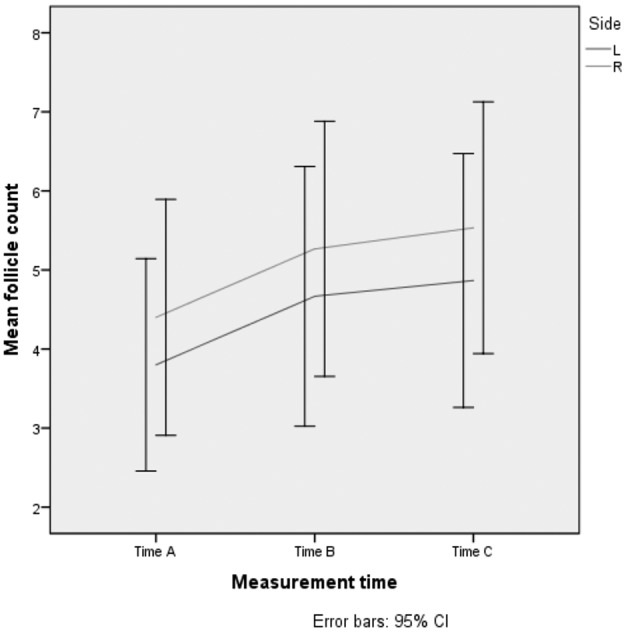
— Progression of the mean follicular diameter (left and right) in 15 patients in whom SOET was performed.

**Figure 3:**
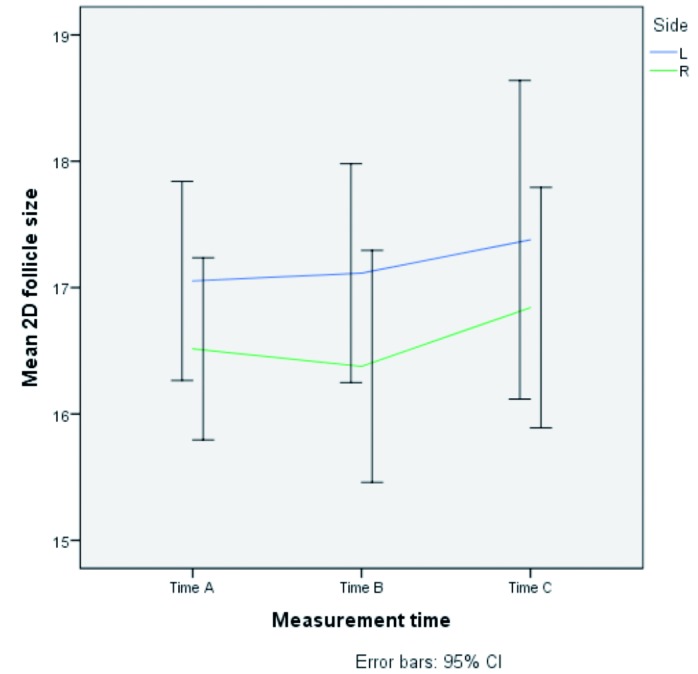
— Progression of mean 2 dimensional follicle size (left and right) in 15 patients in whom SOET was performed

The mean differences in follicular size between the three measurement moments, corrected for side, is statistically not significant (p=0,551) (Fig. 2). Comparing both sides, there is a statistically significant result between the mean follicular size, after correction for measurement moment. (p=0,013). The figure shows the distributions of follicular size for each measurement moment and each side. (Fig. 3)

## Discussion

There is an excellent correlation between the number of follicles when recorded by the patient herself at home (SOET) compared to the number of follicles measured by a physician 12 hours later. This means that SOET is a reliable tool to record the actual count of follicles. There is no statistically significant difference between the mean growth measurements of follicles between different measurement moments. The mean difference on follicle count is very low, so it can be concluded that this will not affect treatment decisions.

This confirms the study results observed by Pereira et al. (2016). This study tested the comparability of SOET with 2D ultrasound performed in the same patient, on the same day and found a high correlation between both (r=0,91) (Pereira et al., 2016). A limitation of this study was the fact that it was the first time the patients used SOET. In our study all patients had used SOET before, they were familiar with the technique. In the study of Pereira et al. (2016), the patients could be biased, because the ultrasound performed by the professional was done just before the SOET ultrasound. In our study the patients performed the SOET ultrasounds like it would be in reality, at home, at their own place and time.

A drawback to this current study is that the patient group was quite small. Ideally this study has to be repeated in a larger series of patients. Only normal responders were included in the study, poor responders will probably be a more challenging group to generate good quality SOET images.

We conclude that our findings indicate that SOET can replace the traditional way of ultrasound monitoring in a well-selected group of patients.

## Declaration

The author report no financial or commercial conflicts of interest.

## References

[1] Cicchetti DV, Sparrow SA (1981). Developing criteria for establishing interrater reliability of specific items: Applications to assessment of adaptive behavior.. Am J Ment Defic.

[2] Gerris J, Delvigne A, Dhont N (2014). Self-operated endo-vaginal telemonitoring versus traditional monitoring of ovarian stimulation in assisted reproduction: an RCT.. Hum Reprod.

[3] Gerris J, Geril A, De Sutter P (2009). Patient acceptance of self- operated endo-vaginal telemonitoring (SOET): proof of concept.. Facts Views Vis Obgyn.

[4] Gerris J, Vandekerckhove F, De Sutter P (2016). Outcome of one hundred consecutive ICSI attempts using patient operated home sonography for monitoring follicular growth.. Facts Views Vis Obgyn.

[5] Magli MC, Van den Abbeel E, Lundin K (2008). Revised guidelines for good practice in IVF laboratories.. Hum Reprod.

[6] La Marca A, Papaleo E, Grisendi V (2012). Development of a nomogram based on markers of ovarian reserve for the individualisation of the follicle-stimulating hormone starting dose in in vitro fertilisation cycles. BJOG.

[7] Pereira I, Von Horn K, Depenbusch M (2016). Self-operated endo- vaginal telemonitoring: a prospective, clinical validation study.. Fertil Steril.

